# Promoter Methylation of *SFRP3* Is Frequent in Hepatocellular Carcinoma

**DOI:** 10.1155/2014/351863

**Published:** 2014-01-21

**Authors:** Ya-Wen Lin, Yu-Lueng Shih, Gi-Shih Lien, Fat-Moon Suk, Chung-Bao Hsieh, Ming-De Yan

**Affiliations:** ^1^Department and Graduate Institute of Microbiology and Immunology, National Defense Medical Center, Taipei, Taiwan; ^2^Graduate Institute of Life Sciences, National Defense Medical Center, Taipei, Taiwan; ^3^Division of Gastroenterology, Department of Internal Medicine, Tri-Service General Hospital, National Defense Medical Center, Taipei, Taiwan; ^4^Division of Gastroenterology, Department of Internal Medicine, Wan Fang Hospital, Taipei Medical University, Taipei, Taiwan; ^5^Division of General Surgery, Department of Surgery, National Defense Medical Center, Tri-Service General Hospital, Taipei, Taiwan

## Abstract

Oncogenic activation of the Wnt/**β**-catenin signaling pathway is common in human cancers. The secreted frizzled-related proteins (SFRPs) function as negative regulators of Wnt signaling and have important implications in carcinogenesis. Because there have been no reports about the role of *SFRP3* in hepatocellular carcinoma (HCC), we investigated the level of methylation and transcription of *SFRP3*. Four HCC cell lines, 60 HCCs, 23 cirrhosis livers, 37 chronic hepatitis livers, and 30 control livers were prescreened for *SFRP3* promoter methylation by methylation-specific polymerase chain reaction (MS-PCR) and bisulfite sequencing. *SFRP3* promoter methylation was observed in 100%, 60%, 39.1%, 16.2%, and 0% in HCC cell lines, primary HCCs, cirrhosis livers, chronic hepatitis livers, and control livers, respectively. Demethylation treatment with 5-aza-2′-deoxycytidine in HCC cells restored or increased the *SFRP3* mRNA expression. We next used quantitative MS-PCR (QMSP) to analyze the methylation level of *SFRP3* in 60 HCCs and their corresponding nontumor tissues. Methylation of *SFRP3* promoter region in HCCs increased significantly compared with control tissues. There is a positive correlation between promoter hypermethylation and *SFRP3* mRNA downregulation. Our data suggest that promoter hypermethylation of *SFRP3* is a common event in HCCs and plays an important role in regulation of *SFRP3* mRNA expression.

## 1. Introduction

Hepatocellular carcinoma (HCC) is the most frequent primary malignancy of the liver and accounts for as many as 1 million deaths annually worldwide [1–5]. The major risk factors include chronic hepatitis B virus (HBV) infection, chronic hepatitis C virus (HCV) infection, environmental carcinogens such as aflatoxin B1 (AFB1), alcoholic cirrhosis, and inherited genetic disorder such as hemochromatosis, Wilson disease, and tyrosinemia. Among them, HBV, HCV, and AFB1 are responsible for approximately 80% of all HCC [1, 2]. Research on molecular genetics and pathogenesis of HCC has become a hot spot in cancer study because of its scientific merits and its clinical importance. Despite rapid expansion of information obtained from these researchers, the molecular mechanism of hepatocarcinogenesis and molecular genetics of HCC remain elusive.

The Wnt/*β*-catenin signaling pathway plays an important role in liver physiology and pathology by regulating a variety of crucial cellular events, including differentiation, proliferation, and survival [6–8]. The Wnt/*β*-catenin pathway can be activated through mutations in *CTNNB1* (encoding *β*-catenin), *AXIN1*, and *AXIN2* [6, 9] in human HCC. The common event is the stabilization of *β*-catenin, which translocates into the nucleus and associates with the T-cell factor (TCF) family of transcription factors for efficient activation of Wnt target genes [10–17]. In addition to genetic mutations, epigenetic changes are also involved in the aberrant activation of Wnt/*β*-catenin signaling pathway in cancer cells [6, 9, 18–22].

Abnormal hypermethylation of CpG islands serves as another mechanism for inactivation of the tumor suppressor gene (TSG) in cancer [23–25]. Hypermethylation of gene promoters has been demonstrated as an early event in hepatocellular carcinogenesis [26–28]. The secreted frizzled-related proteins (SFRPs) function as negative regulators of Wnt signaling and have important implications for carcinogenesis [29]. The secreted frizzled-related protein (SFRP) family plays a significant role in the inhibition of the Wnt signaling pathway in various cancers [30]. The frizzled-related protein (SFRP3) is generally thought to be an inhibitor of Wnt signaling in several cancers [31, 32]. Some reports have demonstrated that SFRP3 has tumor-suppressing activities and could inhibit cell invasiveness in prostate cancer and melanoma cells [31, 32]. However, SFRP3 promotes cell growth, invasion, and inhibition of apoptosis in renal cancer cells [33]. Because there have been no reports about the role of *SFRP3* in hepatocellular carcinoma (HCC), we investigated the level of methylation and transcription of *SFRP3*.

Recently, we have shown that* SFRPs* are often downregulated through promoter hypermethylation in HCC cell lines and clinical HCC tissues [18, 34]. Furthermore, we have demonstrated that restoration of *SFRPs *could attenuate Wnt signaling in HCC cells with *β*-catenin mutation, decrease aberrant accumulation of free *β*-catenin in the nucleus, and then suppress cell growth [34]. We hypothesized that CpG island methylation of the *SFRP3* promoter may play an important role in regulating *SFRP3 *expression in HCC. To test this hypothesis, we used MS-PCR, QMSP, and bisulfite sequencing method to analyze the* SFRP3* methylation pattern in HCCs. The mRNA expression was assessed by quantitative RT-PCR assay. Further, we determined whether treatment of HCC cell lines with a DNA methylation inhibitor, 5-aza-2′-deoxycytidine (5-Aza-CdR), could then restore or increase expression of the *SFRP3 *mRNA.

## 2. Materials and Methods

### 2.1. Tissue Specimens

Sixty paired HCC samples (including HCC tissues, DNA, and RNA samples) and 30 hepatic hemangioma tissues were provided by the Taiwan Liver Cancer Network (TLCN). The TLCN is funded by the National Science Council to provide researchers in Taiwan with primary liver cancer tissues and their associated clinical information. The diagnosis of HCC was confirmed by histology. Experienced pathologist classified the nontumor tissues as chronic hepatitis livers (23 cases) and cirrhosis livers (37 cases). The use of the 60 HCC tissues, paired nontumor parts, and 30 hepatic hemangioma tissues (as control livers) in this study was approved by the Institutional Review Board and the TLCN User Committee.

### 2.2. Cell Lines

We obtained three human HCC cell lines from the American Type Culture Collection (ATCC, Rockville, MD): HepG2, HA22T, Hep3B, and TONG. They were all grown in Dulbecco's modified Eagle's medium (DMEM) supplemented with 10% (w/v) fetal bovine serum, penicillin at 100 U/mL, streptomycin at 100 *μ*g/mL, and L-glutamine at 2 mmol/L (all from Invitrogen, Carlsbad, CA) at 37°C in an atmosphere of 5% (v/v) CO_2_ in air.

### 2.3. 5-Aza-2′-deoxycytidine Treatment

HCC cells were seeded at a density of 1 × 10^5^ cells/100-millimeter dish and allowed to attach for 24 hr. Cells were incubated in 5 µM 5-aza-2′-deoxycytidine (5-Aza-CdR; Sigma Chemical Co., St. Louis, MO) diluted in phosphate-buffered saline (PBS) or in PBS alone for 96 hr to analyze the effect of methylation inhibition on *SFRP3* mRNA expression. All incubations were performed in duplicate dishes, and cells were harvested directly for RNA and DNA isolation.

### 2.4. DNA Extraction

Genomic DNA was extracted from cell lines and tissue samples using a commercial DNA extraction kit (QIAmp Tissue Kit; Qiagen, Hilden, Germany). DNA was isolated according to the manufacturer's protocol.

### 2.5. Bisulfite Modification and Methylation-Specific PCR (MS-PCR)

Genomic DNA isolated from cells and tissue was subjected to bisulfite methylation analysis. We treated DNA with bisulfite using an EZ DNA methylation kit (Zymo Research, Orange, CA) according to the protocol described in the user manual. Briefly, one µg of genomic DNA was denatured by incubation with 0.2 M NaOH. Aliquots of 10 mM hydroquinone and 3 M sodium bisulfite (pH 5.0) were added and the solution was incubated at 50°C for 16 hr. Treated DNA was purified on a Zymo-Spin I column, desulfonated with 0.3 M NaOH, repurified on a Zymo-Spin I column, and resuspended in 20 *μ*L elution buffer. MS-PCR [35] was carried out in a volume of 25 *μ*L containing 1 *μ*L of the sodium-bisulfite-treated DNA with Gold *Taq* DNA polymerase (PE Applied Biosystems, Foster City, CA) as follows. After heating at 92°C for 10 min, PCR was performed in a thermal cycler (GeneAmp 2400, PE Applied Biosystems) for 35 cycles, each of which consisted of denaturation at 92°C for 30 sec, annealing at 61°C for 30 sec, and extension at 72°C for 30 sec, followed by a final 10 min extension at 72°C. The PCR products were analyzed by electrophoresis on a 3% agarose gel. The experiments were repeated three times to ensure reproducibility. The sequences of *SFRP3* promoter, primer, and probes are summarized in Table 1.

### 2.6. Bisulfite Sequencing

Bisulfite-treated genomic DNA was amplified using specific primers for human *SFRP3.* Amplified PCR product was purified and cloned into pCR4-TOPO vector (Invitrogen, Carlsbad, CA). DNA sequencing was performed on at least 5 individual clones using the 377 automatic sequencer (Applied Biosystems, Foster City, CA, USA). The primer sequences and the locations are summarized in Table 1.

### 2.7. Quantitative Methylation-Specific PCR (QMSP)

TagMan-based QMSP (MethyLight) [36] method was used to determine the methylation level of HCCs. We used type II collagen gene (*COL2A*) for an internal reference gene by amplifying the non-CpG sequences. Each sample was analyzed three times. The genomic DNA treatment with M.Sss I methyltransferase (New England Biolabs, Beverly, MA) was used as positive control. The QMSP reactions were done as our previous report [37]. The relative DNA methylation was determined based on the threshold cycles (Ct) of the gene of interest and of the internal reference gene (*COL2A*). The relative DNA methylation level [sample_gene/sample_*COL2A*] was calculated by the ΔCt method [36, 38]. Testing results with Ct-value of *COL2A* greater than 40 were determined as detection failure.

### 2.8. Quantitative RT-PCR

Quantitative RT-PCR analysis was performed on an ABI PRISM 7700 Sequence Detector (Applied Biosystems, Forster City, USA). The match primers and TagMan Probe were obtained from commercial Applied Biosystems Tagman Assay-on Demand Gene Expression products. Glyceraldehyde-3-phosphate dehydrogenase gene (*GAPDH*) was used as an internal control. PCR reaction was carried out using TaqMan PCR master mix reagents kit. Relative gene expression was determined based on the threshold cycles (Ct) of the gene of interest and of the internal reference gene. The mRNA levels of the interest genes were expressed as the ratio of the interest gene to *GAPDH* mRNA for each sample. The level of each interest gene mRNA in each cancer was compared to the level in the corresponding nontumor part [39]. The average Ct value of the *GAPDH *gene was subtracted from the average Ct value of the interest genes for each sample: SFRP3 ΔCt = (Avg. SFRP3 Ct − Avg. GAPDH Ct) and SFRP3 ΔΔCt = (Avg. SFRP3  ΔCt_tumor_ − Avg.  SFRP3  ΔCt_nontumor_). The fold change (2^−SFRP3ΔΔCt^) in expression of the target genes (*SFRP3*) relative to the internal control gene (*GAPDH*) of each analyzed HCC sample was calculated [18, 39].

### 2.9. Statistical Analysis

Associations between methylation of *SFRP3 *and clinical parameters were analyzed by using a chi-square test and Fisher's exact test, where necessary. We correlated the *SFRP3 *methylation status with the liver disease status (control, chronic hepatitis, cirrhosis liver, and HCC) and downregulation of *SFRP3* mRNA expression. Significant differences were analyzed using the paired sample *t*-test or Mann-Whitney *U* test. The significance level was defined as *P* value < 0.05.

## 3. Results 

### 3.1. Hypermethylation of *SFRP3 *Promoter in Primary HCCs

**Figure 1:**
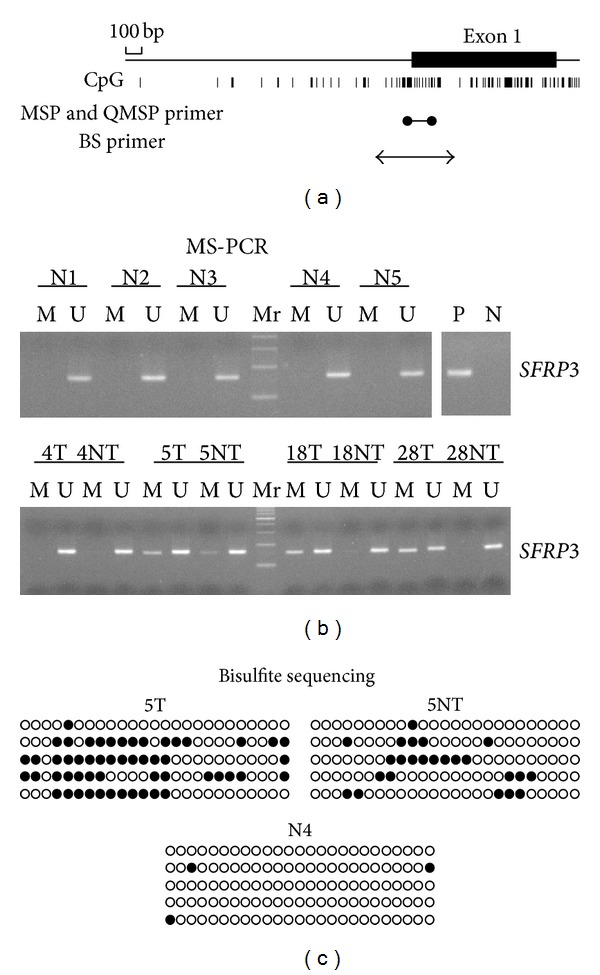
Methylation of *SFRP3* in primary hepatocellular carcinoma tissues. (a) Schematic representation of the promoter region and the first exon of the *SFRP3* gene. The CpG rich areas and the sites of methylation specific PCR (MSP), quantitative MSP, and bisulfite sequencing (BS) primers are indicated. (b) Representative results for four control livers (N1 to N5), four HCCs (T), and their corresponding nontumor livers (NT). Bisulfite-modified genomic DNA was amplified using methylation-specific or unmethylation-specific primer sets. M, methylation-specific PCR product; U, unmethylation-specific PCR. DNA from the peripheral blood lymphocyte (PBL) sample was used as a negative control, and PBL DNA treated with SssI Methylase (New England Biolabs, Beverly, MA) was a positive control. Case numbers are indicated at the top. (c) Summary of bisulfite sequencing. Case numbers of tumors, nontumor tissues, and normal control are indicated at the top. Black and white circles correspond to methylated or unmethylated, respectively.

To investigate the promoter methylation of *SFRP3* in HCC, we first tested for promoter methylation in 30 control livers, 60 primary HCCs, and their corresponding nontumor tissues using MSP (Figures 1(a) and 1(b), Table 2). Aberrant promoter methylation of* SFRP3* gene was observed in 60%, 39.1%, 16.2%, and 0% in primary HCCs, cirrhosis livers, chronic hepatitis livers, and normal controls, respectively. The methylation level within the *SFRP3* promoter was then validated by bisulfite sequencing. Representative results for bisulfite sequencing are shown in Figure 1(c). The CpGs in these regions were frequently methylated in HCC tumors (Figure 1(c), 5T). The methylation of *SFRP3 *promoter was detected in some nontumor parts from HCC patients with chronic hepatitis or cirrhosis (Figure 1(c), 5NT). In contrast, we did not detect promoter hypermethylation in control liver tissues (Figure 1(c), N4). Our data showed that methylation level of* SFRP3 *promoter region in HCCs increased significantly compared with control livers (Table 3).

### 3.2. Promoter Methylation of* SFRP3 *and Downregulation of *SFRP3* mRNA in HCC Cell Lines

**Figure 2:**
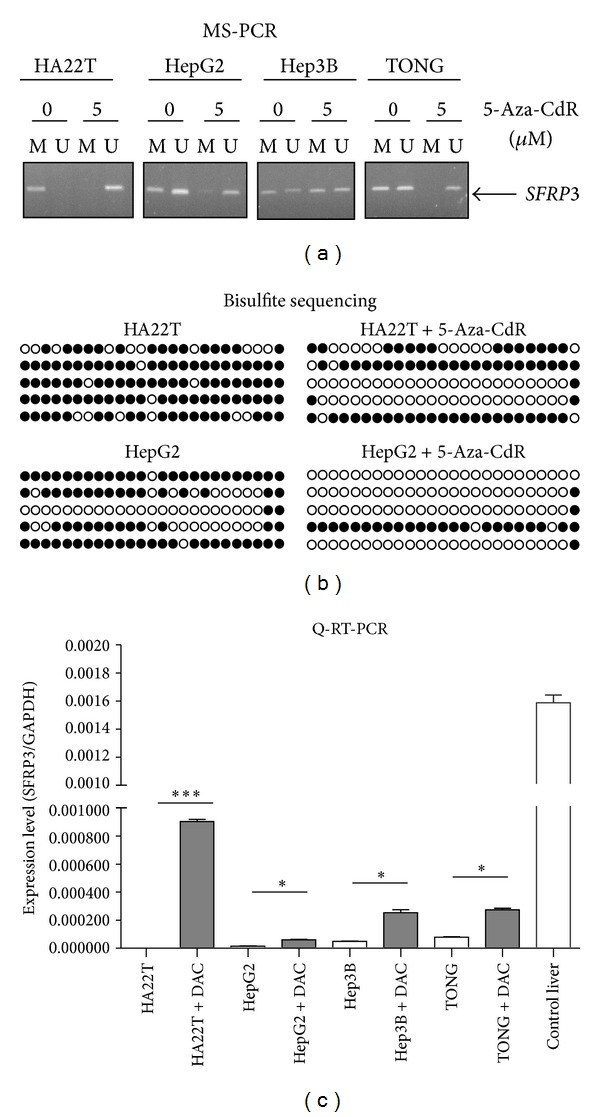
Promoter methylation and downregulation of* SFRP3* in HCC cell lines. (a) Detection of methylation in HCC cell lines using MS-PCR. M, methylation-specific PCR product; U, unmethylation-specific PCR. Four cell lines were treated for 4 days with the indicated concentration of 5-Aza-CdR. MS-PCR assay on DNA isolated from untreated or treated HCC cells. (b) Summary of bisulfite sequencing. The name of HCC cell line is indicated at the top. Black and white circles correspond to methylated or unmethylated, respectively. (c) HCC cell lines were treated with 5-aza-2′-deoxycytidine (5-Aza-CdR, DAC) for 4 days. The mRNA of *SFRP3* was analyzed by Q-RT-PCR. Expression of *GAPDH* was determined as a control for RNA quality. Significant differences were analyzed using the Mann-Whitney *U* test (* for *P* < 0.05 and *** for *P* < 0.001).

We then investigated the methylation level of *SFRP3* promoter in four HCC cell lines (HA22T, HepG2, Hep3B, and TONG) using MSP and bisulfite sequencing. Among four HCC cell lines, our data demonstrated *SFRP3* was fully methylated in HA22T cells and partially methylated in the other cells (Figure 2(a)). Bisulfite sequencing results were summarized in Figure 2(b). The CpGs in these regions was frequently methylated (Figure 2(b)). Quantitative RT-PCR data showed that downregulation of *SFRP3* mRNA in the four HCC lines with* SFRP3* hypermethylation (Figure 2(c)). To confirm that the lack of expression of *SFRP3 *mRNA in the HCC lines was due to promoter hypermethylation, we treated cells with 5-aza-2′-deoxycytidine, an inhibitor of DNA methylation. After treatment with 5 µM of 5-aza-2′-deoxycytidine, the unmethylated promoter DNA was detected by MSP and bisulfite sequencing; *SFRP3* mRNA was restored or increased in the four HCC cell lines (Figures 2(a), 2(b), and 2(c)). These data indicate that hypermethylation of *SFRP3* may be responsible for the absence or downregulation of mRNA transcription.

### 3.3. Downregulation of *SFRP3 *mRNA Is Correlated with Promoter Methylation in Primary HCCs

**Figure 3:**
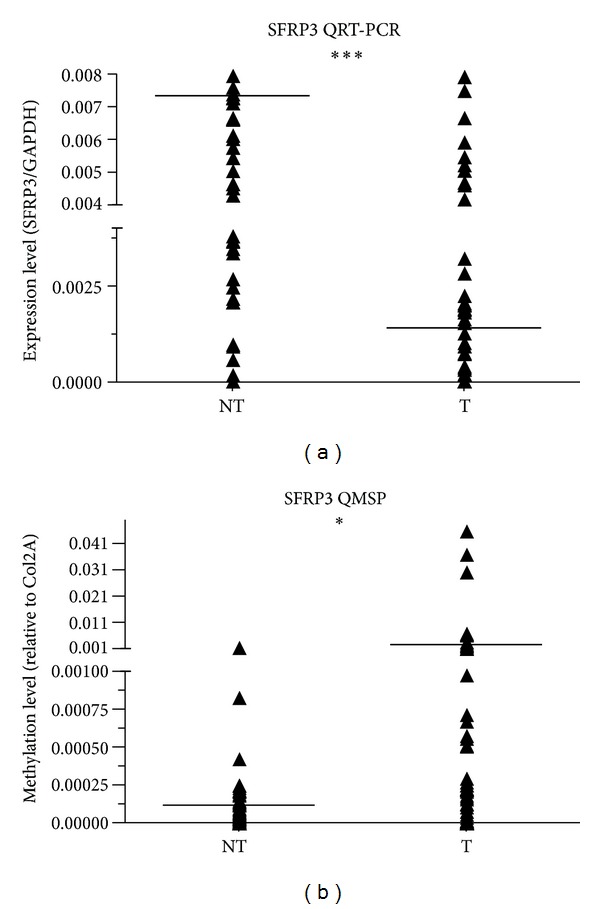
Frequent downregulation of *SFRP3* is associated with promoter hypermethylation in primary HCCs. The *SFRP3* transcripts of 60 primary HCCs (T) and their corresponding adjacent nontumor tissues (NT) were analyzed by RT-PCR and normalized to the internal control (*GAPDH*). Next, the methylation status of clinical HCC tissues was checked by QMSP and normalized to the internal reference gene *COL2A*. Significant differences were analyzed using the paired sample *t*-test or Mann-Whitney *U* test (* for *P* < 0.05 and *** for *P* < 0.001).

To study the relation between *SFRP3* promoter methylation level and *SFRP3* mRNA expression, we first checked the mRNA level of 60 primary HCCs and their corresponding adjacent nontumor tissues by quantitative RT-PCR. Our data showed *SFRP3* mRNA expression was significantly downregulated in the primary HCCs as compared with the adjacent nontumor tissues (*P* < 0.0001) (Figure 3(a)). Next, we checked the methylation status of the HCC cell lines and clinical HCC tissues by QMSP. Hypermethylation was confirmed in the HCC tissues compared with the nontumor liver tissues (*P* < 0.01) (Figure 3(b)). In 36 of 60 HCCs (60%), *SFRP3* mRNA was significantly downregulated (by >2-fold, Table 4). There was a statistically significant association between the downregulation of *SFRP3* mRNA and the methylation status of *SFRP3 *in HCCs (35/36 versus 17/24 resp.; *P* < 0.01) (Table 4). There were some HCCs without methylation; however, their *SFRP3 *mRNA expression were downregulated.

## 4. Discussion

Here we demonstrate that *SFRP3* is significantly hypermethylated and downregulated in HCCs when compared with control livers and nontumor livers (containing chronic hepatitis or cirrhosis livers) (*P* < 0.0001, Table 3 and Table 2). *SFRP3* mRNA expression could be restored or increased after HCC cells treatment with a DNA methyltransferase (DNMT) inhibitor, 5-aza-2′-deoxycytidine (Figure 2). We found a significant correlation between methylation and transcription level in primary tissues (Table 4, *P* < 0.001). In accordance with our data, promoter methylation has been detected in chronic hepatitis tissue and cirrhosis liver tissues, indicating that DNA methylation may be an early event in the pathogenesis of HCC [19, 40]. Put together, our data suggest that that downregulation of* SFRP3* mRNA through promoter hypermethylation is an early event during carcinogenesis and may be involved in the aberrant activation of Wnt/*β*-catenin signaling in HCC. Moreover, *SFRP3* mRNA was downregulated more than twofold in the absence of promoter hypermethylation in 71% of HCCs (17 of 24) (Table 4). The decreased *SFRP3* mRNA level might be due to genetic changes or other epigenetic changes like histone modification.

Our data suggest that promoter hypermethylation of *SFRP3* is a common event in HCCs and plays an important role in regulation of *SFRP3* mRNA expression. Therefore epigenetic regulation of the Wnt/*β*-catenin pathway has been implicated as a possible therapeutic target in human cancer. Further investigations are required to explore the importance of *SFRP3* in the development of hepatocellular carcinoma.

## 5. Conclusions

In conclusion, promoter hypermethylation of *SFRP3* is a frequent event in HCCs and epigenetic downregulation of *SFRP3* mRNA may contribute to aberrant activation of Wnt/*β*-catenin in HCC. This is the first report about hypermethylation and downregulation of *SFRP3* mRNA in HCC.

## Figures and Tables

**Table 1:** The primer and probe sequences used in this study.

Primer sequence (5′-3′)	Primer name	Assay
GTGTTGTTTTGGGGTTTTGTATTTGTATG	SFRP3 UF	MSPCR
CTACCTCCCACCTAAAAAAAAACTCCAC	SFRP3 UR	MSPCR
TTGGGGTGGGTTTTTTAGTGAGGGGT	BS01 F	BS sequencing
AACAAAAAAAACRCTCAAAAAAAACC	BS01 R	BS sequencing
GGCGTTGTTTTGGGGTTTCGTATTC	SFRP3 MF	MSPCR, QMSP
TCCCGCCTAAAAAAAAACTCCG	SFRP3 MR	MSPCR, QMSP
CTCTACCCTCCAATACC	probe	QMSP
TCCCGAGGCCATCGTTACT	SFRP3 F	QRT-PCR (SyBr)
AGGCTTACATTTACAGCGTTCAC	SFRP3 R	QRT-PCR

Sequence of *SFRP3* promoter:

aaaaaaaaagtccaagtgtattagagctgttagtttccacgttaacccttaaggagcaaagctcaagagttctaattccactaggtggggggggcgggaatagaaggaaaaaaccccttttccttgcttctggtggcctatttgtagtcat

gaacagcatttctttgtttctctctctctttttttttttttttaaaggcaatcctccccccacctcctcccccgcagttattgaaaatggagacctctgtagtcactagctctgggttgatatggctccaccgttgctcgcaggggtctgtgttttccg

ctacttggacaaagtgacattgcttaagcctttccccccaccaggtctgactttctgcagagccagtgattgcagaggaaaagctgtagtttgcttaaaggaaatacctccaggaaggagggtctcgggtgggttcccaagtggggaact

agggggacttttccgtagggaattggggtgggctcttcagtgaggggctaggggctcgtttctggggccaaagacgggttccctagtgtgagggcgcgctcgactcggcgctgtcttggggtctcgcactcgcacggcttcgcaccccac

cgcctgcgactcccaggccttctcttccccgggcgcccactccattctcgggaagagcagccggcactggagggcagagactgccccaggggcggagctccctctcaggcgggaggtaggaaagtgcagagccgcccgggcagagg

cacagacgtccctgcggggctcctcctgagcgtccctcctgccagccagggtcgcagccgcagcggcggccgcagctcttagcccacacaggacttgtaaactcttactgcacccttctctcccattaggagcttttcctccctccttccc

cccaacccctctgtcctcctcactttggggaatttaatgctttctttagcatctttttgtgtgcgtgatctaggggaggagacaccccagagctccaactagctctcagctgaattctacttttgtttttatgtcttcctcgcctcctctcgtgtcc

ctcttatctgactgatctgcgaagtctgatgcttctgccagagggagaggaataaatagatgttgctgcttccgaaggcttagacGTTGGGAAAGAGCAGCCTGGGCGGCAGGGGCGGTGGCTG

GAGCTCGGTAAAGCTCGTGGGACCCCATTGGGGGAATTTGATCCAAGGAAGCGGTGATTGCCGGGGGAGGAGAAGCTCCCAGATCCTTGTG

TCCACTTGCAGCGGGGGAGGCGGAGACGGCGGAGCGGGCCTTTTGGCGTCCACTGCGCGGCTGCACCCTGCCCCATCCTGCCGGGATC.

**Table 2:** *SFRP3* mRNA expression in primary HCCs by relative quantitative RT-PCR.

Patient no.	*SFRP3* methylation	ΔCt	ΔΔCt	*SFRP3* tumor part
		*SFRP3-GAPDH *	ΔCt tumor − ΔCt nontumor	Rel. to nontumor
1T	U	9.03	1.68	0.3121
1NT	U	7.35		

2T	M	10.05	2.81	0.1426
2NT	M	7.24		

3T	U	7.63	−0.47	1.3851
3NT	U	8.10		

4T	U	7.58	−0.51	1.4191
4NT	U	8.09		

5T	U	11.54	0.82	0.5684
5NT	U	10.72		

6T	U	5.92	−0.29	1.2226
6NT	U	6.21		

7T	M	7.40	1.38	0.3856
7NT	U	6.03		

8T	U	15.00	6.10	0.0146
8NT	U	8.91		

9T	M	8.95	1.91	0.2661
9NT	U	7.04		

10T	M	9.03	1.79	0.2892
10NT	M	7.24		

11T	M	15.00	9.03	0.0019
11NT	M	5.97		

12T	M	9.10	1.35	0.3923
12NT	M	7.75		

13T	U	9.62	1.58	0.3356
13NT	U	8.04		

14T	U	6.27	−0.71	1.6358
14NT	U	6.98		

15T	M	15.00	7.90	0.0042
15NT	U	7.10		

16T	M	15.00	7.14	0.0071
16NT	M	7.86		

17T	M	9.34	1.13	0.4569
17NT	M	8.21		

18T	U	5.10	−1.01	2.0069
18NT	U	6.11		

19T	M	6.75	1.04	0.4863
19NT	M	5.71		

20T	U	15.00	7.87	0.0043
20NT	U	7.14		

21T	M	15.00	8.13	0.0036
21NT	U	6.87		

22T	U	9.92	3.48	0.0899
22NT	U	6.45		

23T 23NT	M M	9.05 7.63	1.42	0.3737

24T	M	8.47	1.24	0.4248
24NT	M	7.23		

25T	M	6.96	0.61	0.6552
25NT	M	6.35		

26T	U	5.14	0.11	0.9298
26NT	U	5.04		

27T	M	12.37	5.31	0.0253
27NT	M	7.06		

28T	M	15.00	6.21	0.0136
28NT	U	8.80		

29T	U	5.67	2.49	0.1780
29NT	U	3.18		

30T	M	9.23	1.44	0.3680
30NT	U	7.79		

31T	U	15.00	6.34	0.0123
31NT	U	8.66		

32T	U	8.28	1.18	0.4429
32NT	U	7.11		

33T	U	12.14	5.96	0.0161
33NT	U	6.18		

34T	U	7.63	2.47	0.1811
34NT	U	5.16		

35T	M	6.98	4.65	0.0398
35NT	M	2.33		

36T	M	15.00	5.01	0.0310
36NT	M	9.99		

37T	U	15.00	9.40	0.0015
37NT	U	5.61		

38T	M	15.00	10.72	0.0006
38NT	M	4.28		

39T	M	7.90	1.10	0.4665
39NT	U	6.80		

40T	M	15.00	7.48	0.0056
40NT	U	7.52		

41T	M	8.97	1.22	0.4308
41NT	U	7.75		

42T	M	9.25	2.17	0.2222
42NT	U	7.08		

43T	U	15.00	8.77	0.0023
43NT	U	6.23		

44T	M	8.76	0.22	0.8586
44NT	M	8.54		

45T	M	8.92	2.86	0.1377
45NT	U	6.06		

46T 46NT	U U	10.34 8.17	2.17	0.2222

47T	M	15.00	9.13	0.0018
47NT	U	5.88		

48T	U	15.00	9.16	0.0018
48NT	U	5.85		

49T	M	15.00	9.43	0.0014
49NT	U	5.57		

50T	M	15.00	6.78	0.0091
50NT	U	8.22		

51T	M	15.00	6.16	0.0140
51NT	U	8.85		

52T	M	15.00	10.77	0.0006
52NT	U	4.24		

53T	U	11.21	3.77	0.0733
53NT	U	7.44		

54T	M	12.27	10.28	0.0008
54NT	U	1.99		

55T	M	15.00	4.94	0.0326
55NT	U	10.06		

56T	U	7.74	1.98	0.2535
56NT	U	5.76		

57T	U	10.39	3.52	0.0872
57NT	U	6.87		

58T	M	15.00	9.01	0.0019
58NT	U	5.99		

59T	M	11.36	3.99	0.0632
59NT	U	7.38		

60T	M	7.52	2.55	0.1713
60NT	U	4.97		

NT: nontumor part; T: tumor part; M: methylated; U: unmethylated.

The range given for *SFRP3* tumor part relative to nontumor part is determined by evaluating the expression: 2^−ΔΔCt^.

**Table 3:** Frequency of *SFRP3* promoter methylation in 30 control livers and 60 paired HCC and adjacent nontumor tissue samples.

Diagnosis	No. of cases with *SFRP3* methylation	*P* value
Control livers* (*n* = 30)	0 (0%)	<0.0001
Chronic hepatitis (*n* = 37)	6 (16.2%)	
Cirrhosis (*n* = 23)	9 (39.1%)	
HCC (*n* = 60)	36 (60%)	

*Thirty control tissues were from 30 hepatic hemangiomas. Statistical analysis was determined by chi-square test.

**Table 4:** Statistical correlation between *SFRP3* mRNA expression and methylation status of *SFRP3* CpG island in HCCs.

	Methylation of CpG island (no. of cases)	No methylation of CpG island (no. of cases)	*P* value
Downregulation of *SFRP3* ≥ twofold			
Present	35	17	*P* < 0.01
Absent	1	7	

## Abbreviation

HCC: Hepatocellular carcinoma
SFRP3: Secreted frizzled-related protein 3
5-Aza-CdR: 5-Aza-2′-deoxycytidine
MSP: Methylation-specific PCR
RT-PCR: Reverse transcription-polymerase chain reaction
HBV: Hepatitis B virus
HCV: Hepatitis C virus
TSG: Tumor suppressor gene.

## References

[1] Bosch FX, Ribes J, Cléries R, Díaz M (2005). Epidemiology of hepatocellular carcinoma. *Clinics in Liver Disease*.

[2] Bosch FX, Ribes J, Borràs J (1999). Epidemiology of primary liver cancer. *Seminars in Liver Disease*.

[3] Befeler AS, Di Bisceglie AM (2002). Hepatocellular carcinoma: diagnosis and treatment. *Gastroenterology*.

[4] El-Serag HB (2004). Hepatocellular carcinoma: recent trends in the United States. *Gastroenterology*.

[5] El-Serag HB, Mason AC (1999). Rising incidence of hepatocellular carcinoma in the United States. *The New England Journal of Medicine*.

[6] Thompson MD, Monga SPS (2007). WNT/*β*-catenin signaling in liver health and disease. *Hepatology*.

[7] Chiba T, Zheng Y-W, Kita K (2007). Enhanced self-renewal capability in hepatic stem/progenitor cells drives cancer initiation. *Gastroenterology*.

[8] Zeng G, Apte U, Cieply B, Singh S, Monga SPS (2007). siRNA-mediated *β*-catenin knockdown in human hepatoma cells results in decreased growth and survival. *Neoplasia*.

[9] Behari J (2010). The Wnt/*β*-catenin signaling pathway in liver biology and disease. *Expert Review of Gastroenterology and Hepatology*.

[10] Cadigan KM, Nusse R (1997). Wnt signaling: a common theme in animal development. *Genes and Development*.

[11] Logan CY, Miller JR, Ferkowicz MJ, McClay DR (1999). Nuclear *β*-catenin is required to specify vegetal cell fates in the sea urchin embryo. *Development*.

[12] Polakis P (2000). Wnt signaling and cancer. *Genes and Development*.

[13] Yost C, Torres M, Miller JR, Huang E, Kimelman D, Moon RT (1996). The axis-inducing activity, stability, and subcellular distribution of *β*-catenin is regulated in Xenopus embryos by glycogen synthase kinase 3. *Genes and Development*.

[14] Behrens J, von Kries JP, Kühl M (1996). Functional interaction of *β*-catenin with the transcription factor LEF- 1. *Nature*.

[15] Cui J, Zhou X, Liu Y, Tang Z, Romeih M (2003). Wnt signaling in hepatocellular carcinoma: analysis of mutation and expression of beta-catenin, T-cell factor-4 and glycogen synthase kinase 3-beta genes. *Journal of Gastroenterology and Hepatology*.

[16] de La Coste A, Romagnolo B, Billuart P (1998). Somatic mutations of the *β*-catenin gene are frequent in mouse and human hepatocellular carcinomas. *Proceedings of the National Academy of Sciences of the United States of America*.

[17] Farazi PA, DePinho RA (2006). Hepatocellular carcinoma pathogenesis: from genes to environment. *Nature Reviews Cancer*.

[18] Shih Y-L, Shyu R-Y, Hsieh C-B (2006). Promoter methylation of the secreted frizzled-related protein 1 gene SFRP1 is frequent in hepatocellular carcinoma. *Cancer*.

[19] Caldwell GM, Jones C, Gensberg K (2004). The Wnt antagonist sFRP1 in colorectal tumorigenesis. *Cancer Research*.

[20] Sarrió D, Moreno-Bueno G, Hardisson D (2003). Epigenetic and genetic alterations of APC and CDH1 genes in lobular breast cancer: relationships with abnormal E-cadherin and catenin expression and microsatellite instability. *International Journal of Cancer*.

[21] Satoh S, Daigo Y, Furukawa Y (2000). AXIN1 mutations in hepatocellular carcinomas, and growth suppression in cancer cells by virus-mediated transfer of AXIN1. *Nature Genetics*.

[22] Suzuki H, Gabrielson E, Chen W (2002). A genomic screen for genes upregulated by demethylation and histone deacetylase inhibition in human colorectal cancer. *Nature Genetics*.

[23] Herman JG, Baylin SB (2003). Gene silencing in cancer in association with promoter hypermethylation. *The New England Journal of Medicine*.

[24] Herman JG, Merlo A, Mao L (1995). Inactivation of the CDKN2/p16/MTS1 gene is frequently associated with aberrant DNA methylation in all common human cancers. *Cancer Research*.

[25] Esteller M, Sparks A, Toyota M (2000). Analysis of adenomatous polyposis coli promoter hypermethylation in human cancer. *Cancer Research*.

[26] Yu J, Zhang HY, Ma ZZ, Lu W, Wang YF, Zhu JD (2003). Methylation profiling of twenty four genes and the concordant methylation behaviours of nineteen genes that may contribute to hepatocellular carcinogenesis. *Cell Research*.

[27] Yang B, Guo M, Herman JG, Clark DP (2003). Aberrant promoter methylation profiles of tumor suppressor genes in hepatocellular carcinoma. *American Journal of Pathology*.

[28] Schagdarsurengin U, Wilkens L, Steinemann D (2003). Frequent epigenetic inactivation of the RASSF1A gene in hepatocellular carcinoma. *Oncogene*.

[29] Suzuki H, Watkins DN, Jair K-W (2004). Epigenetic inactivation of SFRP genes allows constitutive WNT signaling in colorectal cancer. *Nature Genetics*.

[30] Finch PW, He X, Kelley MJ (1997). Purification and molecular cloning of a secreted, Frizzled-related antagonist of Wnt action. *Proceedings of the National Academy of Sciences of the United States of America*.

[31] Ekström EJ, Sherwood V, Andersson T (2011). Methylation and loss of secreted frizzled-related protein 3 enhances melanoma cell migration and invasion. *PLoS ONE*.

[32] Zi X, Guo Y, Simoneau AR (2005). Expression of Frzb/secreted Frizzled-related protein 3, a secreted Wnt antagonist, in human androgen-independent prostate cancer PC-3 cells suppresses tumor growth and cellular invasiveness. *Cancer Research*.

[33] Hirata H, Hinoda Y, Ueno K, Majid S, Saini S, Dahiya R (2010). Role of secreted frizzled-related protein 3 in human renal cell carcinoma. *Cancer Research*.

[34] Shih Y-L, Hsieh C-B, Lai H-C (2007). SFRP1 suppressed hepatoma cells growth through Wnt canonical signaling pathway. *International Journal of Cancer*.

[35] Herman JG, Graff JR, Myöhänen S, Nelkin BD, Baylin SB (1996). Methylation-specific PCR: a novel PCR assay for methylation status of CpG islands. *Proceedings of the National Academy of Sciences of the United States of America*.

[36] Ogino S, Kawasaki T, Brahmandam M (2006). Precision and performance characteristics of bisulfite conversion and real-time PCR (MethyLight) for quantitative DNA methylation analysis. *Journal of Molecular Diagnostics*.

[37] Lai H-C, Lin Y-W, Huang R-L (2010). Quantitative DNA methylation analysis detects cervical intraepithelial neoplasms type 3 and worse. *Cancer*.

[38] Coleman WB, Rivenbark AG (2006). Quantitative DNA methylation analysis: the promise of high-throughput epigenomic diagnostic testing in human neoplastic disease. *Journal of Molecular Diagnostics*.

[39] Livak KJ, Schmittgen TD (2001). Analysis of relative gene expression data using real-time quantitative PCR and the 2-ΔΔCT method. *Methods*.

[40] Kondo Y, Kanai Y, Sakamoto M, Mizokami M, Ueda R, Hirohashi S (2000). Genetic instability and aberrant DNA methylation in chronic hepatitis and cirrhosis—a comprehensive study of loss of heterozygosity and microsatellite instability at 39 loci and DNA hypermethylation on 8 CpG islands in microdissected specimens from patients with hepatocellular carcinoma. *Hepatology*.

